# Automated group assignment in large phylogenetic trees using GRUNT: GRouping, Ungrouping, Naming Tool

**DOI:** 10.1186/1471-2105-8-402

**Published:** 2007-10-18

**Authors:** Daniel Dalevi, Todd Z DeSantis, Jakob Fredslund, Gary L Andersen, Victor M Markowitz, Philip Hugenholtz

**Affiliations:** 1 Biological Data Management and Technology Center, Lawrence Berkeley National Laboratory, 1 Cyclotron Road, Berkeley, CA, 94720, USA; 2 Center for Environmental Biotechnology, Lawrence Berkeley National Laboratory, 1 Cyclotron Road, Berkeley, CA, 94720, USA; 3 Bioinformatics Research Center, University of Aerhus, Høgh-Guldbergs Gade 10, Building 090, DK-8000 Århus C, Denmark; 4 Microbial Ecology Program, DOE Joint Genome Institute, 2800 Mitchell Dr., Walnut Creek, CA 94598, USA

## Abstract

**Background:**

Accurate taxonomy is best maintained if species are arranged as hierarchical groups in phylogenetic trees. This is especially important as trees grow larger as a consequence of a rapidly expanding sequence database. Hierarchical group names are typically manually assigned in trees, an approach that becomes unfeasible for very large topologies.

**Results:**

We have developed an automated iterative procedure for delineating stable (monophyletic) hierarchical groups to large (or small) trees and naming those groups according to a set of sequentially applied rules. In addition, we have created an associated ungrouping tool for removing existing groups that do not meet user-defined criteria (such as monophyly). The procedure is implemented in a program called GRUNT (GRouping, Ungrouping, Naming Tool) and has been applied to the current release of the Greengenes (Hugenholtz) 16S rRNA gene taxonomy comprising more than 130,000 taxa.

**Conclusion:**

GRUNT will facilitate researchers requiring comprehensive hierarchical grouping of large tree topologies in, for example, database curation, microarray design and pangenome assignments. The application is available at the greengenes website [1].

## Background

Phylogenetic trees are a standard way to visualize and interpret homologous sequences, such as for the delineation of taxonomies. With the explosion of sequence data, trees are becoming large and unwieldy. Nowhere is this more apparent than with the small subunit ribosomal RNA (16S rRNA) gene, one of the most widely accepted marker genes for global phylogenies [2] and one of the cornerstones of our present understanding of evolutionary biology [3]. Currently there are in excess of 150,000 full-length 16S sequences in public repositories with the number increasing rapidly. A fully expanded tree comprising 150,000 sequences is nearly impossible to navigate. One solution to this problem is to collapse (ideally) monophyletic sets of sequences into groups. ARB [4] was one of the earliest tools providing the ability to collapse and expand groups of sequences to facilitate tree navigation. ARB allows curators to add or remove groups manually, but as the 16S database expands manual group curation is no longer feasible, especially since trees have a dynamic structure due to frequent updates. We have developed a tool, GRUNT, to automate this step that includes grouping, ungrouping, and naming functions. The tool is implemented as part of the Greengenes database [5], but is also available as a standalone tool.

It should be noted that GRUNT is not a de novo clustering method, but rather a tool that identifies clusters already present in pre-existing tree topologies. This means that methods, such as large-scale Bayesian [6] and maximum likelihood [7,8] inference, able to incorporate assumptions such as rate-heterogeneity, can be used as the basis for GRUNT. Consequently, GRUNT is a tool that facilitates rapid objective classification of hierarchical monophyletic groups in the absence of formal classification, which is lagging behind particularly for environmental clone sequences.

## Implementation

GRUNT was written to specifically interface with the ARB software [4] to facilitate group curation of greengenes.arb, the ARB database from which the Greengenes taxonomy is extracted. GRUNT exploits a number of features of ARB including the ability to display multiple fields in any order at the terminal nodes in a tree and the ability to export trees from ARB in XML format that include terminal node fields, branch length, bootstrap values and any existing group names. We found, however, that ARB is lacking an XML to Newick [9] converter necessary for re-importing GRUNT-annotated XML trees. Therefore we built our own converter for this purpose [10].

A step-by-step protocol on how to export and re-import ARB trees with the appropriate fields is provided at the Greengenes website ([1]; see Additional File 1). However, the program can be applied to XML trees generated by any software provided the format fulfills the ARB XML schema. Sample XML files are provided through the greengenes website ([1]; see Additional File 2).

## Results and Discussion

### Defining new groups

GRUNT defines new groups in XML trees based on up to four parameters; branch length (mL), bootstrap support values (mS) and number of daughter taxa circumscribed by the branch under consideration (mC). These parameters can be user-defined and the settings represent minimum (threshold) values. mL and mS are used to help ensure that only reproducibly monophyletic groups are defined. Although bootstrap values are typically used to infer monophyly, we include branch length as an option (proxy) for defining monophyly since it may not be feasible to bootstrap very large trees. These parameters can be used independently or together (both mL and mS must be satisfied for the group to be defined if used together). Minimum number of taxa in a group, mC, is included to allow iterative hierarchical and nested assignment of groups (see below).

**Figure 1:**
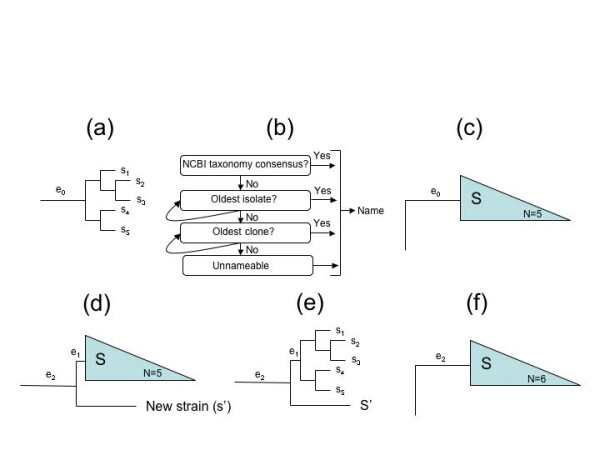
Curation of groups using GRUNT before and after database updates. (a) An ungrouped tree with interior nodes labeled s_1 _to s_5 _and parent branch e_0_. The grouping function (addG) identifies that e_0 _satisfies a minimum branch length (mL) and/or bootstrap support (mS) and that the group contains at least a minimum number of taxa (mC). (b) The naming rules (see text) are applied and the group-name S is proposed and recorded in the XML file. (c) The name is assigned to the newly formed group. (d) New sequences are added to an existing tree as part of an update, and a new sequence, s', is placed basal to group S. (e) The ungrouping function (rmvG) removes groups with branch-lengths below mL and/or mS, in this example e_1 _is not supported and the group S is removed. (f) The grouping and naming tools are then reapplied and identify the new stable parent branch e_2 _which then reforms the group S. Note that the name for group S may not be the same as in 1d depending on the taxon composition of the newly formed group.

GRUNT traverses the tree by starting at any of the leaves separated from the root by the greatest number of bifurcations, and walks towards the root. Once encountering a branch that satisfies mL and/or mS and mC, it creates a new group (Fig. 1) and assigns it a name (see below) provided that the group does not contain any predefined subgroups. It then repeats the procedure for the next leaf separated from the root by the highest number of bifurcations. If during the walk it encounters an existing group before identifying a candidate branch for a new group, it moves onto the next leaf. This prevents over-grouping of the tree. The program terminates when all leaves have been examined.

Since GRUNT only assigns groups to existing trees and does not create or alter topologies, it is dependent on the accuracy of the tree to which it is applied. For example, if a set of sequences is misaligned against a reference alignment resulting in an incorrect placement as a separate lineage in a tree, GRUNT may assign a group name to the spurious lineage.

### Naming new groups

Prior to creating names for newly defined groups, GRUNT creates a dictionary of all existing names in an input tree. As new names are assigned, they are added to the dictionary. This is to ensure that every group has a unique name by not reusing existing names. Names are based on 5 fields commonly associated with sequence records and exported from greengenes.arb; i) unique identifier, ii) sequence type (clone or isolate), iii) NCBI taxonomy, iv) submission date and v) clone or organism name. For other ARB 16S rRNA databases, such as silva [11], equivalent taxon fields would need to be identified and exported. The requirement for multiple taxon fields was the reason that XML was chosen over the more standard Newick format, which can only hold one taxon field.

New group names are derived from the five taxon fields by applying a set of sequential naming rules based on members of the group under consideration:

1. Name the group based on the consensus of the taxon name (e.g. *Pseudomonas*) of the lowest NCBI rank after removing taxonomically uninformative records from consideration (see below). In the unlikely event that two or more taxon names are equally represented in the group, GRUNT will concatenate the names.

2. If the consensus name has already been taken, use the organism name of the oldest isolate record that does not contain any interfering characters (see below).

3. If the oldest isolate record name has already been taken, use the next oldest isolate record lacking interfering characters, repeat as necessary.

4. If no isolates are present or have been excluded due to other rules (name already in use or contains interfering characters), use the name of the oldest clone that does not contain any interfering characters.

5. If the oldest clone record name has already been taken, use the next oldest clone record lacking interfering characters, repeat as necessary.

6. If all naming options are exhausted, label group UNNAMEABLE followed by a numerical code based on the computer system clock.

Uninformative or redundant field strings are excluded from the naming schema by applying a set of forbidden names that cannot be part of a group name. These include "environmental sample", "unclassified", "uncultured" "unidentified", "cluster" and "isolate". Users can add to this list by means of editing a text file (see Additional File 3). In the same file, characters that may interfere with other tree reading or parsing programs can similarly be excluded from group names, these include any names beginning with an integer or "nan" (interpreted as not a number) and the following characters: "!@#$%^&*().,". Newly defined group names are written to the *groupname *parameter of the relevant branch tag in the XML file (see Additional Files 1 &2).

This naming schema ensures that every group defined by GRUNT will have a unique name, and that names should be relevant for the taxa that they encompass. However, as with many automated annotation tools, GRUNT facilitates but does not replace manual taxonomic classification of records. GRUNT can also be applied to XML trees with a single taxon field, whereby the group name is either chosen randomly from the group member names, or as a consensus of the member names. Note that the unique group name constraint also applies in this case.

### Iteration and performance

The tree-traversing rule that prevents groups from being formed if an existing group is encountered prevents over-grouping, but also means that only small peripheral groups will be formed if a small group size is used. Therefore, GRUNT is most effective for comprehensive but conservative nested grouping if a large mC is initially chosen, such as 1000, and the process *iterated *in decrements of mC. This also has the benefit that larger groups will have preferential naming over smaller groups due to the unique group name constraint. A perlscript that runs GRUNT iteratively is available on the website ([1]; see Additional File 4).

**Figure 2:**
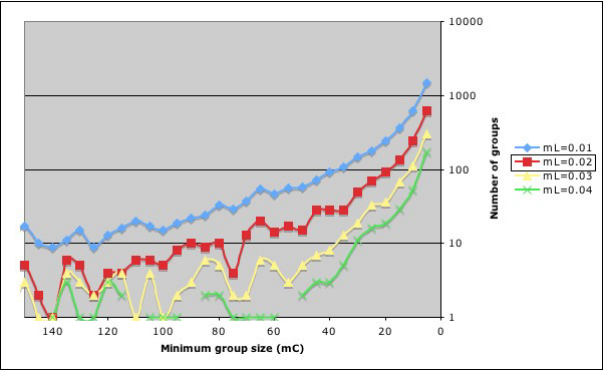
Number of newly defined groups when iterating minimum group size (mC) from 1000 to 5 in decrements of 5 for four minimum branch lengths (mL). Only groups from 150 to 5 are shown for clarity. A non-linear scale is used for the Y-axis to highlight differences in assignments for large groups (missing data points mean that no groups were assigned for that iteration). The total number of defined groups for these settings was 4197, 1582, 699 and 356 for 0.01, 0.02, 0.03 and 0.04 respectively. The default mL setting for grouping is 0.02 (boxed).

GRUNT takes ~25 seconds per cycle to run on the ~130,000 taxa Greengenes tree using a MacBook Pro (2.33 GHz Intel Core 2 Duo with 3 GB 667 MHz DDR2 SDRAM). Running 200 iterative cycles from mC 1000 to 5 in decrements of 5 produced between 356 to 4197 new groups depending on branch length stringency. Fig. 2 shows the number of groups created per cycle for a selection of mC values for four settings of minimal branch length. As expected, number of groups assigned increases as branch length (monophyly stringency) decreases, and number of groups assigned increases as group size decreases.

### Removal of groups

Since curators often dedicate much effort to manually annotating group names, GRUNT, by default, will not modify existing names. However, trees are not static entities due to the rapid database expansion and require constant revision, which was the original impetus for developing GRUNT. Hence, we also developed an ungrouping tool that parses an existing topology looking for groups that are highly unlikely to represent monophyletic lineages and removes them prior to the grouping/naming cycle. A simple example is illustrated in Fig. 1. The addition of a new sequence, s', subdivides the branch, e_0_, leading to a previously monophyletic group S (Fig. 1c&d). Group S is identified by the ungrouping tool based on either a branch length or bootstrap value (e_1 _in Fig. 1d) that falls below a user-defined minimum threshold. The default branch length setting for ungrouping is 0.002. There are, however, exceptions to this rule where the user may not want to remove a group under any circumstances. These holy groups can be specified by the user in a text file supplied to GRUNT before running the removal step (see Additional File 5).

## Conclusion

GRUNT enables the iterative hierarchical assignment of groups and group names to phylogenetic trees according to a set of rules that can be partly defined by the user. This can result in hundreds to thousands of group assignments for large trees (>100,000 taxa) that improve subsequent tree navigation, and facilitate the ability to identify incorrectly placed taxa. Assigning a large number of groups is beyond the ability of manual curators, particularly when databases are regularly updated that necessitates regrouping of tree topologies. The ungrouping function of GRUNT is necessary for the updating process as new taxa can disrupt previously defined groups (Fig. 1). It should be noted, however, that GRUNT only does the grunt work and is not a replacement for manual curation.

Although GRUNT was developed specifically to streamline curation of the Greengenes taxonomy, it may be useful for any application where a comprehensive hierarchical clustering of large tree topologies is required. For example, the interpretation of the recently published comprehensive 16S rRNA PhyloChip microarray [12,13], is optimized if the Greengenes taxonomy (upon which it is based) is accurately and densely grouped. Experimental PhyloChip data can be browsed using heat maps where grouped rows correspond to a set of taxa whose population dynamics correlate across multiple arrays [14,15]. In the past, recognition of phylogenetic relationships among heat map rows has been difficult. Now, as each taxon is annotated with accurate group taxonomy, visual linking of phylogenetically near neighbors is facilitated. Another possible application is the selection of organisms (and their common genes) for defining pan-genomes, "the global gene repertoire of a bacterial species [16]" at the species level and all higher taxonomic ranks. This requires an accurate phylogeny of organisms with sequenced genomes, with all monophyletic groups being comprehensively assigned. As the number of sequenced genomes goes from the hundreds to the thousands in the coming years it will no longer be possible to perform manual grouping, necessitating automated methods such as GRUNT.

## Availability and requirements

***Project name***: GRUNT

***Project home page***: 

***Operating system***: platform independent, source code available at project home page ([1]; see Additional File 6)

***Programming language***: C++

***Licence***: GNU GPL

## Authors' contributions

PH and DD planned and executed the project and wrote the manuscript, TZD integrated GRUNT into the greengenes website, JF wrote the XML to Newick converter and VMM and GLA supported the project. All authors read and approved the final manuscript.

## Supplementary Material

Additional file 1 GRUNT how to notes. step by step instructions on the use of GRUNT Click here for file

Additional file 2 sample XML file. an example of an XML file that is used as the input tree file for the grouping and ungrouping functions in GRUNT Click here for file

Additional file 3 GRUNT forbidden words and characters. an example text file of words and (toxic) characters that are removed from consideration when naming new groups by the GRUNT grouping function Click here for file

Additional file 4 GRUNT iteration perl script. a perl script for iterating the group naming procedure in GRUNT proceeding from larger to smaller groups Click here for file

Additional file 5 GRUNT holy names. an example text file of group names that cannot be deleted by the GRUNT ungrouping function Click here for file

Additional file 6 GRUNT source code. compressed directory containing the source code for GRUNT Click here for file
