# Supplementary material for: Automated group assignment in large phylogenetic trees using GRUNT: GRouping, Ungrouping, Naming Tool
Source: BMC Bioinformatics. 2007 Oct 18;8:402. doi: 10.1186/1471-2105-8-402 (PMC2228325; doi:10.1186/1471-2105-8-402)

1) *preparing and exporting tree_all as an XML tree from greengenes.arb using a GRUNT-specific configuration*

1.1) confirm that no records have data in both clone and organism field, GRUNT expects only one of these fields to contain data

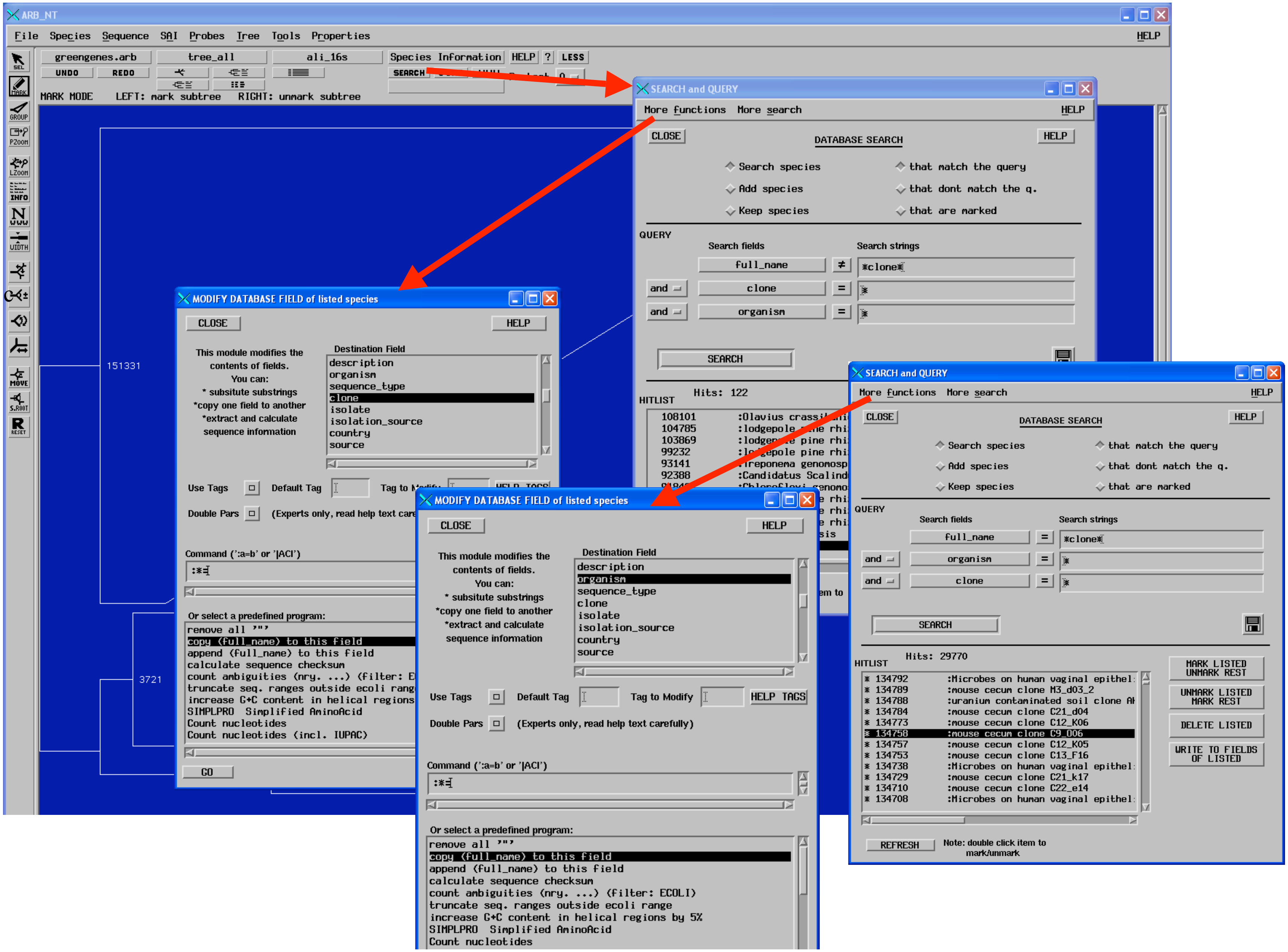

1.2) check that current all records have submit_date information, add a future date to records lacking info

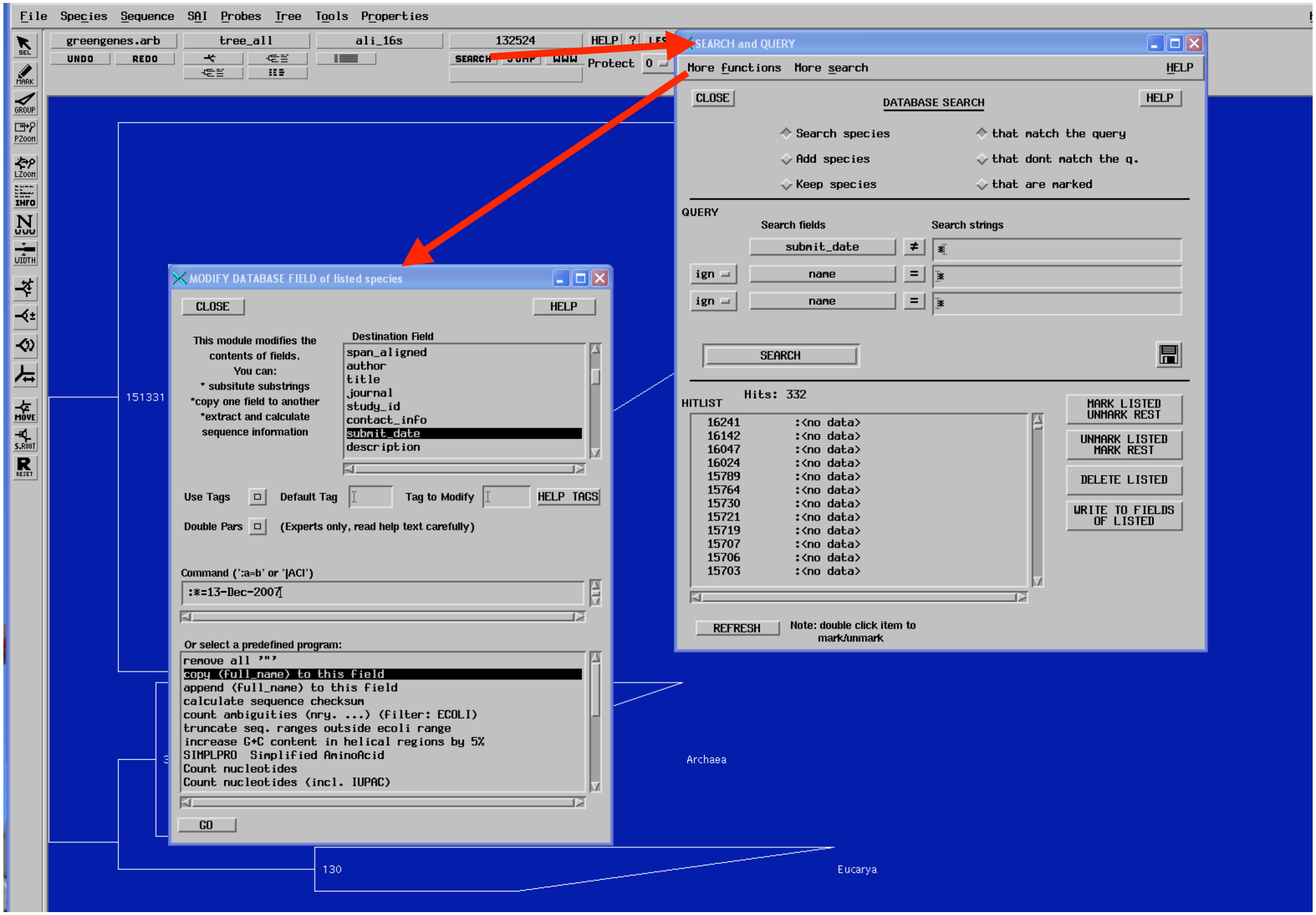

1.3) set NDS configuration to GRUNT setting

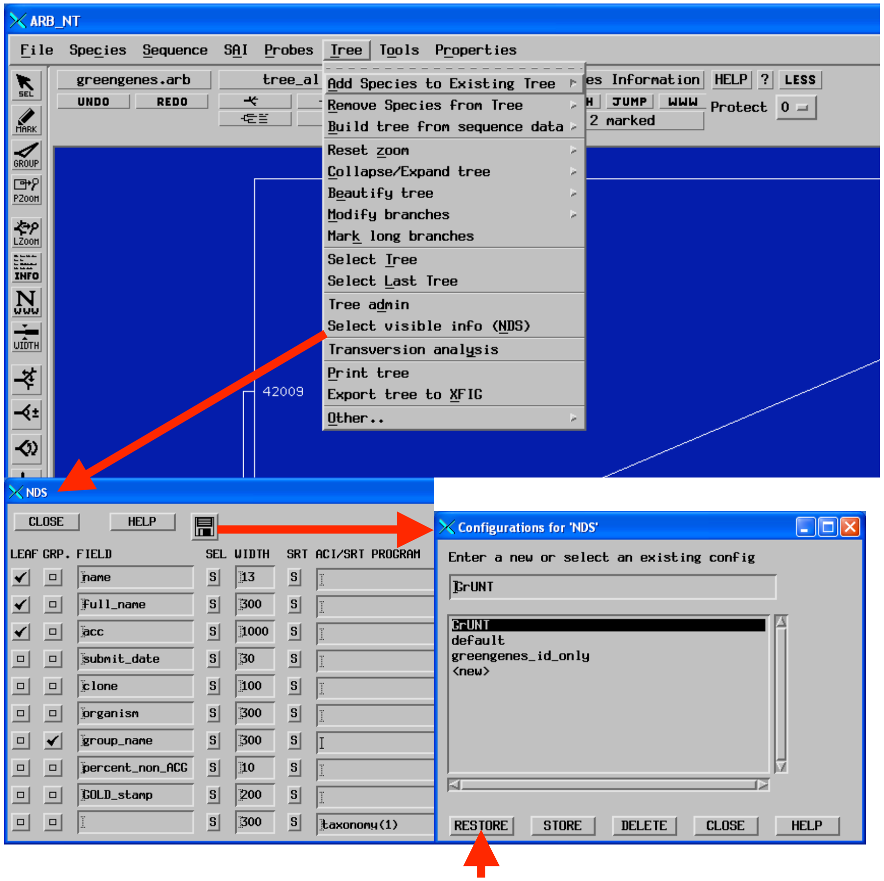

1.4) export tree_all in XML format

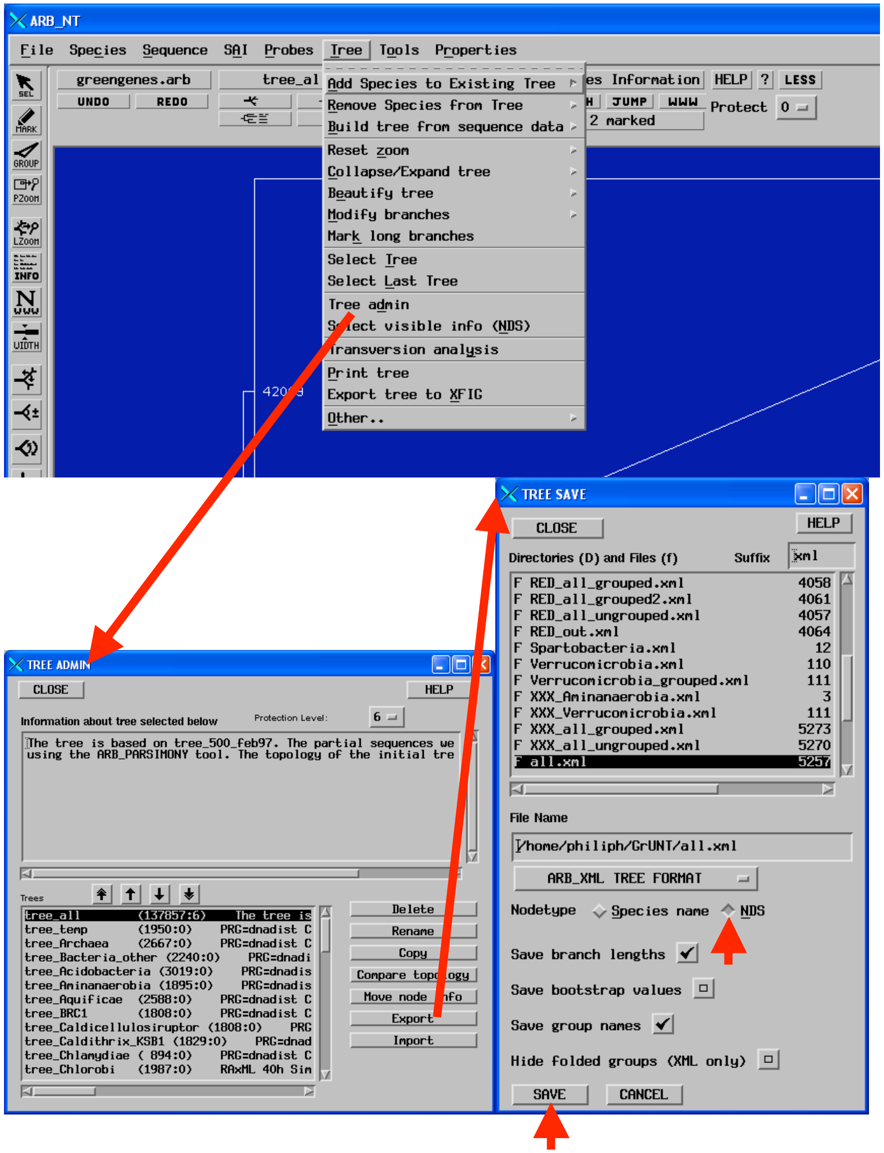

2) *preparing GRUNT-associate files*

2.1) modify forbidden_names.txt and holy_names.txt as necessary

```
  GNU nano 2.0.2             File: forbidden_names.txt                    Modified

# This file contains words that groupnames cannot include
# Only used in the -rmvG option
# All words are separated with new line.
# Currently added are:

environmental sample
unclassified
uncultured
unidentified
cluster
isolate
et al
:BEGIN:NUM:

# There is also a set of toxic characters that groupnames cannot
# have. These are also used in -addG option as opposed to the
# forbidden patterns. Defined them here (no separator between!):

TOXIC: ()/\<>+^$%#@!&

^G Get Help   ^O WriteOut   ^R Read File  ^Y Prev Page  ^K Cut Text   ^C Cur Pos
^X Exit       ^J Justify    ^W Where Is   ^V Next Page  ^U UnCut Text ^T To Spell
```

```
  GNU nano 2.0.2              File: holy_names.txt

Deltaproteobacteria
Firmicutes

^G Get Help   ^O WriteOut   ^R Read File  ^Y Prev Page  ^K Cut Text   ^C Cur Pos
^X Exit       ^J Justify    ^W Where Is   ^V Next Page  ^U UnCut Text ^T To Spell
```

2.2) run GRUNT help file to see all parameters

```
gojiro:~/GrUNT> ./GrUNT -h
----------------------------------------------------------------------
GrUNT - Grouping /  Ungrouping Namming Tool
Version (1,0,20), last update 30 May 2007
A software for automatic assignment of groupnames to 16S trees
maintained by ARB and Greengenes
Written by: Daniel Dalevi
----------------------------------------------------------------------
Syntax:
./GrUNT -f tree.xml <options>
----------------------------------------------------------------------
Type of analysis:
-addG:  Adding groupnames.
-rmvG:  Removing groupnames.
-showG: Will print groupnames  of tree in file "-f" to the output file "-o"
-shLS:  Same as showG but will output branch lengths with support values.
Options:
-f:   In file in ARB's xml formate.
-o:   Out file             (out.xml, or out.txt if "-shLS")
-lf:  Log file             (log.txt)
-hf:  "Holy names". File with groupnames that cannot be removed
      from tree. Entries are listed as rows, one by one.
      If no file is specified, no groups names are "holy"
      Another file will also be produced called RED_*.xml
-pf:  Pattern file
      File with specific words that a groupname cannot contain.
      The option is used when removing groups. For example,
      if specifying "XXX", all groupnames that contain this
      pattern will be removed.
-mC:  Minimum count of taxa in group (10)
-mxC: Maximum count of taxa in group. Negative means inactive (-1)
-mL:  Minimum branch length, a negative number means inactive (0.02)
-mS:  Minimum bootstrap support, a negative number means inactive (-1)
----------------------------------------------------------------------
Example syntax:
./GrUNT -addG -f Cyano.xml -o out.xml -mC 10 -mL 0.001 -mS 75
./GrUNT -rmvG -f Cyano_isolates.xml -pf notallowed.txt -mL 0.01
./GrUNT -showG -f Cyano_isolates.xml -o group_list.txt
./GrUNT -shLS -f Cyano_isolates.xml -o lengths_and_support.txt
----------------------------------------------------------------------
gojiro:~/GrUNT>
```

3) *running GRUNT*

3.1) use the ungroup function to remove unwanted groups in tree_all

```
gojiro:~/GrUNT> ./GrUNT -rmvG -f all.xml -o all_ungrouped.xml -pf forbidden_names.txt -hf holy_names.t
xt -mL 0.002 -lf ungroup_log.txt



--------------------------------------------------
The GrUNT has removed 149 old group(s)
Each group has:
(i) Branch length < 0.002
(ii) Branch support < -1
Input file: all.xml
Output file: all_ungrouped.xml
Reduced file: RED_all_ungrouped.xml
Forbidden pattern file: forbidden_names.txt
Holy file: holy_names.txt

This is version (1,0,19)
Latest update was made in 30 April 2007.
For help, type, "GrUNT -h"
--------------------------------------------------
gojiro:~/GrUNT>
```

3.2) modify the GRUNT perlscript parameters

```
#! /usr/bin/perl -w
use strict;

my $file = shift;
my $out_file = shift;
my $work_file = "XXX_".$file;


system( "cp $file $work_file" );

# define run cycle parameters here:
my $max_group_size = 1000;
my $min_group_size = 5;
my $group_size_decrement = 1;
my $branch_length = 0.02;

for( my $i = $max_group_size; $i >= $min_group_size; $i = $i-$group_size_decrement )
{
    print "Running iteration: $i\n";
    my $err = system( "./GrUNT -addG -f $work_file -pf toxic_names.txt -o $out_file -mC $i[illegible]
    system( "cp $out_file $work_file" );
}
```

3.3) run the group function via the perlscript

```
gojiro:~/GrUNT> perl GrUNT_cycle.pl all_ungrouped.xml all_grouped.xml > group_log.txt
```

3.4) rerun ungrouping function on output file as a cross check

```
gojiro:~/GrUNT> ./GrUNT -rmvG -f all_grouped.xml -o all_groupedXchecked.xml -pf forbidden_names.
txt -hf holy_names.txt -mL 0.002 -lf ungroupXcheck_log.txt
--------------------------------------------------
The GrUNT has removed 0 old group(s)
Each group has:
(i) Branch length < 0.002
(ii) Branch support < -1
Input file: all_grouped.xml
Output file: all_groupedXchecked.xml
Reduced file: RED_all_groupedXchecked.xml
Forbidden pattern file: forbidden_names.txt
Holy file: holy_names.txt

This is version (1,0,19)
Latest update was made in 30 April 2007.
For help, type, "GrUNT -h"
--------------------------------------------------
gojiro:~/GrUNT> arb
Using ARBHOME='/usr/local/arb'
Please wait while the program ARB is starting .....
gojiro:~/GrUNT> ARB: Loading '.arb_prop/ntree.arb'
ARB: Loading '/home/philiph/.arb_prop/ntree.arb' done

ARB: Loading '/home/philiph/greengenes.arb'
ARB: no FastLoad File '/home/philiph/greengenes.ARM' found: loading entire database
```

3.5) convert the reduced* XML output file to Newick

```
gojiro:~/GrUNT> python greengeneXML2newick.py RED_all_groupedXchecked.xml
wrote RED_all_groupedXchecked.xml.newick
leaves: 137857
internal nodes: 66450
Traceback (most recent call last):
  File "greengeneXML2newick.py", line 156, in ?
    items.append(int(node.getAttribute('itemname')))
ValueError: invalid literal for int(): Cn2Glab3
```

Reduced files only use the greengenes identifier to label taxa, this is a requirement for the xml2newick converter

4) *loading GRUNT tree back into greengenes.arb*

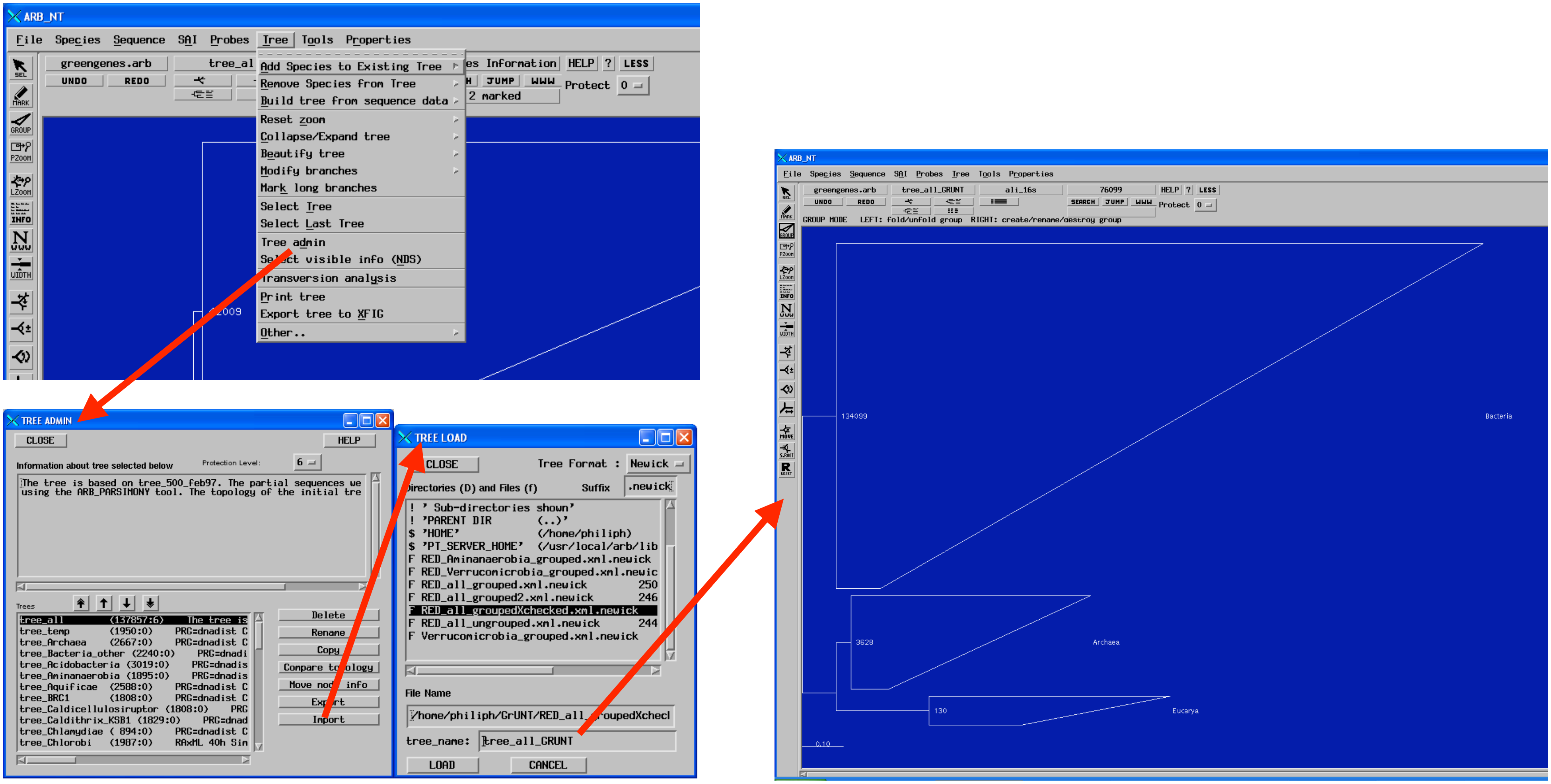

Supplement: Additional file 1 — GRUNT how to notes. step by step instructions on the use of GRUNT [file 1471-2105-8-402-S1.pdf]
