# Supplementary material for: Automated group assignment in large phylogenetic trees using GRUNT: GRouping, Ungrouping, Naming Tool
Source: BMC Bioinformatics. 2007 Oct 18;8:402. doi: 10.1186/1471-2105-8-402 (PMC2228325; doi:10.1186/1471-2105-8-402)
Supplement: Additional file 5 — GRUNT holy names. an example text file of group names that cannot be deleted by the GRUNT ungrouping function [file 1471-2105-8-402-S5.pdf]

Deltaproteobacteria
Firmicutes
